# Prognosis of nontuberculous mycobacterial pulmonary disease according to the method of microbiologic diagnosis

**DOI:** 10.1038/s41598-021-87197-9

**Published:** 2021-04-13

**Authors:** Sooim Sin, Seungchul Han, Yeon Joo Lee, Young-jae Cho, Jong Sun Park, Ho Il Yoon, Choon-Taek Lee, Jae Ho Lee

**Affiliations:** 1 Department of Internal Medicine, Kangwon National University Hospital, Chuncheon-si, Gangwon-do Republic of Korea; 2 College of Medicine, Kangwon National University, Chuncheon-si, Gangwon-do Republic of Korea; 3 Division of Pulmonary and Critical Care Medicine, Department of Internal Medicine, Seoul National University Hospital, Seoul, Republic of Korea; 4 Department of Radiology, Seoul National University College of Medicine, Seoul, Republic of Korea; 5 Division of Pulmonary and Critical Care Medicine, Department of Internal Medicine, Seoul National University College of Medicine, Seoul National University Bundang Hospital, Bundang-gu, Seongnam-si, Gyeonggi-do Republic of Korea

**Keywords:** Diseases, Medical research

## Abstract

Microbiological criteria for nontuberculous mycobacterial pulmonary disease (NTM-PD) require cultures from two separate sputum or one non-sputum specimen. However, there is limited data on the progression of NTM-PD following non-sputum culture-based diagnosis. We compared the disease progression of NTM-PD diagnosed with non-sputum vs sputum cultures. We included 833 patients and divided them into sputum NTM isolation (n = 123), sputum NTM-PD (n = 558), and non-sputum NTM-PD groups (n = 152). Disease progression, defined as radiographic aggravation and therapy initiation, was compared between groups. The median observation time was 60.5 months (interquartile range, 31.4–96.0). The non-sputum NTM-PD group showed longer treatment-free survival (log-rank test; p = 0.009) and lower risk of treatment (adjusted hazard ratio [aHR] of sputum NTM-PD group, 1.36; 95% confidence interval (CI), 1.01–1.84) than the sputum NTM-PD group. The non-sputum NTM-PD group showed longer radiographic aggravation-free survival (Log-rank test; p = 0.015) and lower risk of radiographic aggravation (aHR of sputum NTM-PD group, 1.52; 95% CI, 1.06–2.19) than the sputum NTM-PD group. NTM-PD diagnosed using methods other than sputum culture showed a low risk of disease progression and progressed slower than NTM-PD diagnosed from a sputum culture. NTM-PD diagnosed using methods other than sputum culture may be a mild disease, not equivalent to NTM-PD diagnosed from sputum culture.

## Introduction

Since 2000, the prevalence and mortality of nontuberculous mycobacterial pulmonary disease (NTM-PD) have continuously increased worldwide^1–3^. Therefore, appropriate diagnosis and treatment of NTM-PD is becoming more important. However, NTM-PD is difficult to diagnose owing to its broad and indolent clinical features. According to the 2007 official American Thoracic Society (ATS) and Infectious Diseases Society of America (IDSA) statements for diagnosis of NTM-PD, microbiologic diagnosis of NTM-PD is typically based on positive results from at least two separate sputum cultures. A single isolation of NTM from expectorated sputum is not considered sufficient for a definitive microbiologic diagnosis because NTM is ubiquitous^4,5^. Alternatively, if the sputum culture is negative or patients are unable to produce sputum, NTM-PD diagnosis can be made when bronchoscopic specimens or lung tissue cultures are positive^4^. Those who meet all the diagnostic criteria, including microbiological criteria, can be considered for initiation of treatment.

Patients with more than one positive sputum culture for NTM exhibited a more typical clinical course of pulmonary disease compared to those with only one positive sputum culture^6^. The diagnostic yield of bronchoscopic specimens for NTM-PD has been investigated in several studies, suggesting its superior sensitivity over sputum culture^7–10^. However, no study has yet examined whether the disease progression of patients diagnosed with NTM-PD with NTM isolated from bronchoscopic specimens or lung tissue is equivalent to that of patients diagnosed using sputum culture. Thus, the microbiological criteria for NTM-PD are based on low-quality evidence, and it remains to be clarified whether NTM-PD diagnosed following microbiological criteria other than sputum culture can provide a proper prognosis for pulmonary disease. In this study, we compared the prognosis of NTM-PD diagnosed using bronchoscopy or lung tissue with the prognosis of NTM-PD diagnosed using sputum culture and with a single positive NTM isolation.

## Methods

### Study design and population

This was a retrospective cohort study based on the NTM cohort at Seoul National University Bundang Hospital, which enrolled 1531 patients with NTM isolated from specimens at least once between January 2005 and December 2016. The time of NTM isolation was defined as time zero. Patients with suspected NTM-PD, based on symptoms or radiologic findings, were recommended for spontaneous sputum expectoration at least twice for microbiological diagnosis. Patients who showed insufficient results on sputum specimens and those who were not able to expectorate sputum underwent additional bronchoscopy if NTM-PD was highly suspected. We designated patients with a single NTM isolation from sputum for 2 years from time zero as the sputum NTM isolation group. Patients whose NTM was isolated at least twice from separately expectorated sputum samples for 2 years from time zero were designated as the NTM-PD group diagnosed using sputum culture (sputum NTM-PD group). Finally, patients with positive results from bronchoscopic specimens [e.g., bronchial washing or bronchoalveolar lavage (BAL)] or lung tissue for 2 years from time zero were designated as the NTM-PD group diagnosed using other methods (non-sputum NTM-PD group). Patients with a single NTM isolation from several sputum specimens and positive results from the bronchoscopic specimens were assigned to the non-sputum NTM-PD group.

Patients without baseline chest computed tomography (CT) or follow-up chest CT and those with a total follow-up period of less than 1 year were excluded. Patients who had a history of NTM isolation or received treatment for NTM-PD prior to enrollment in the current study were excluded. Additionally, patients diagnosed with active pulmonary tuberculosis within 1 year before or after NTM isolation were excluded (in the sputum NTM-PD group, the time of the second positive sputum culture was considered the time of NTM isolation). Patients diagnosed with lung malignancies or interstitial lung disease during the follow-up period and those with solitary nodules on baseline radiography were excluded. Finally, we included only patients with isolated NTM of the *Mycobacterium avium* complex, *Mycobacterium abscessus* complex, and *Mycobacterium kansasii*.

**Figure 1:**
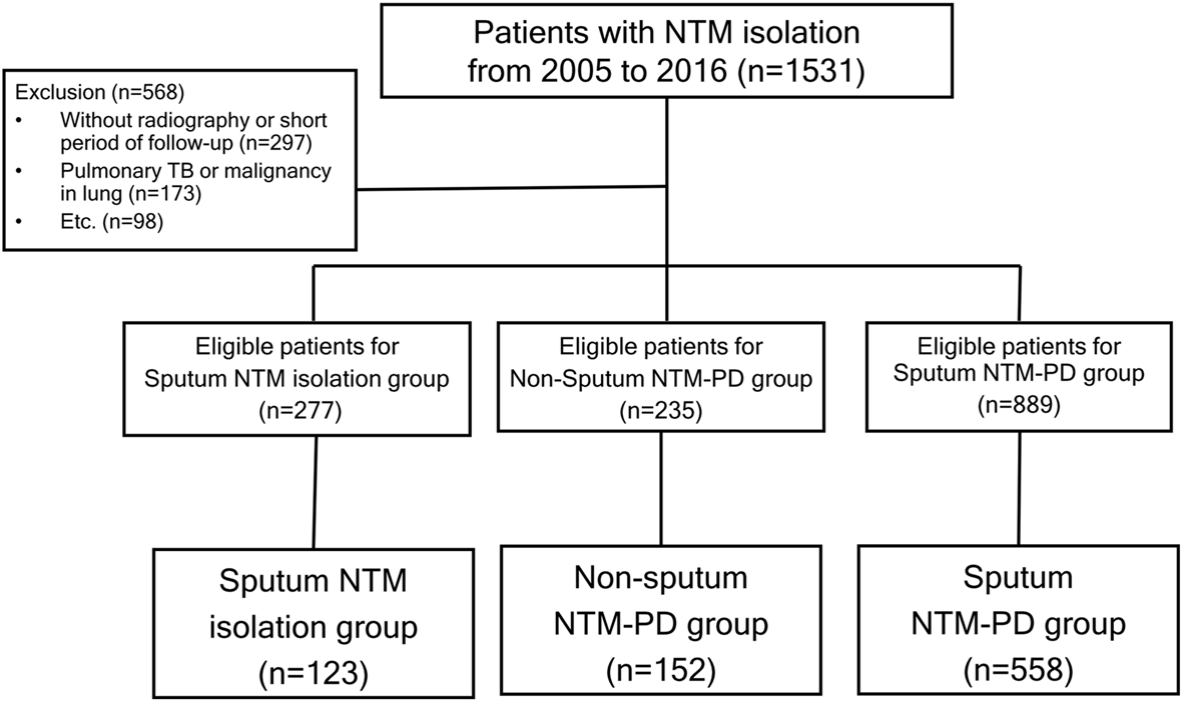
Flow diagram of the study population enrolled from NTM cohort at Seoul National University Bundang Hospital, from January 2005 to December 2016. *NTM *nontuberculous mycobacterium, *NTM-PD* nontuberculous mycobacterial pulmonary disease, *TB* tuberculosis. Patients with a single NTM isolation from sputum were designated as the sputum NTM isolation group, patients with positive results from bronchoscopic [e.g., bronchial washing or bronchoalveolar lavage (BAL)] or lung tissue specimens were designated as the NTM-PD group diagnosed using other methods (non-sputum NTM-PD group) and patients with NTM isolation at least two times from separately expectorated sputum samples were designated as the NTM-PD group diagnosed using sputum culture (sputum NTM-PD group). Etc. includes those who had a history of NTM isolation or received treatment for NTM-PD or diagnosed with interstitial lung disease during the follow-up period or those with solitary nodules on baseline radiography or those with Mycobacterium other than *avium* complex, *abscessus* complex, and *kansassi.*

Of the patients in the cohort, 277 were classified into the sputum NTM isolation group, 235 into the non-sputum NTM-PD group, and 934 into the sputum NTM-PD group. Among eligible patients, 704 were excluded based on the exclusion criteria. Accordingly, 123 patients in the sputum NTM isolation group, 152 in the non-sputum NTM-PD group, and 558 in the sputum NTM-PD group were analyzed (Fig. 1).

This study was approved by the Institutional Review Board of the Seoul National University Bundang Hospital (IRB No. B-1812-511-102) and conformed to the tenets of the Declaration of Helsinki. Informed consent was waived by the Institutional Review Board due to the retrospective nature of the study.

### Data collection and assessment

All results of acid-fast bacilli (AFB) staining and cultures from January 2005 to December 2016 were reviewed. Baseline demographics including age, sex, comorbidities, smoking status, and respiratory symptoms within 3 months before or after time zero were collected. Medical records, including prescriptions during the follow-up period, were collected from the electronic medical record system for review. Chest CT images performed during the follow-up period were also collected. Baseline radiographic features including radiographic type, extent of the disease (number of lobes with disease), presence of cavity, presence and severity of bronchiectasis (assessed using a modified Reiff score which measuring the number of lobes with bronchiectasis and severity of bronchial dilatation) were assessed based on baseline chest CT images^11,12^. Radiographic type was categorized as fibrocavitary, nodular bronchiectatic, and unclassifiable according to previously described methods^4,13,14^.

### Assessment of disease progression

To compare prognoses between groups, radiographic aggravation and initiation of treatment for NTM-PD were assessed. Each patient was radiographically evaluated at regular intervals during outpatient clinic follow-ups (usually followed by serial X-ray at 3–4 month intervals and chest CT at one-year intervals), or when patients complained of symptoms worsening. All chest CT scans were reviewed for evaluation of radiographic aggravation. NTM-PD-related radiographic features such as presence, severity, and extent of bronchiectasis, cavity, nodules, and consolidation, and the presence of infiltrates were assessed according to the ATS/IDSA guidelines and previous studies^4,15,16^. Radiographic aggravation was assessed independently by a radiologist and a pulmonologist, blinded to the clinical data. Any discrepancies were addressed, and final decision was reached by consensus. We identified patients who began treatment for NTM-PD after time zero, regardless of the treatment period. The proportion of patients with radiographic aggravation and treatment initiation were compared between the groups. The time to radiographic aggravation and treatment initiation were also compared between the groups. Moreover, disease progression risk according to the diagnostic methods was estimated after adjustment for variables.

### Statistical analyses

One-way analysis of variance (ANOVA) was used for comparison of demographics involving continuous variables, and the Chi-squared test and Bonferroni’s correction for multiple comparisons were used to compare demographics involving categorical variables. Kaplan–Meier analysis with the log-rank test was performed to compare outcomes between groups. In addition, post-hoc analysis on the log-rank test with Bonferroni’s correction was performed for multiple comparisons of survival curves. Cox proportional hazard regression analysis was used to calculate adjusted hazard ratios (aHR) and 95% confidence intervals (CIs). *P*-values lower than 0.05 were considered statistically significant. Data analysis was conducted using STATA 13 software (Stata Corp, College Station, TX, USA).

## Results

### Baseline characteristics and characteristics of isolated NTM

The median observation time was 60.5 months (IQR, 31.4–96.0) for all patients, 70 months (IQR, 33.0–109.0) for the sputum NTM isolation group, 87.9 months (IQR, 58.3–121.0) for the sputum NTM-PD group, and 58.0 months (IQR, 28.0–105.6) for the non-sputum NTM-PD group). Among the 152 patients in the non-sputum NTM-PD group, 133 (87.5%) had a positive culture from bronchial washing, 12 (7.9%) had a positive culture from BAL, and 7 patients (4.6%) had a positive culture from percutaneous needle aspiration of lung tissue.

Baseline characteristics of the subjects and characteristics of isolated NTM are shown in Table 1. Patients in the sputum NTM-PD group had a lower body mass index (BMI) than the sputum NTM isolation group (p = 0.017). Patients in the non-sputum NTM-PD group were younger than those in the sputum NTM-PD group (p = 0.001). Patients in the non-sputum NTM-PD group had fewer complaints of cough or sputum than the other groups. The sputum NTM-PD group included more patients with bronchiectasis than the other groups. There were no significant differences in baseline characteristics, including proportion of sex, smoking status, history of pulmonary tuberculosis, comorbidities (other than chronic obstructive pulmonary disease), respiratory symptoms of dyspnea, and weight loss between the groups. Patients in the sputum NTM-PD group were more likely to have fibrocavitary-type disease, more extensive lobar involvement, and cavitary lesions than the other groups. Moreover, patients with sputum NTM-PD were more likely to have a positive AFB smear. *Mycobacterium avium* complex was the most frequently isolated strain in all groups, followed by *Mycobacterium intracellulare* and *Mycobacterium abscessus* complex.

**Table 1:** Baseline characteristics and clinical features of the patients.

Variables	Sputum NTM isolation group (n = 123)	Non-sputum NTM-PD group (n = 152)	Sputum NTM-PD group (n = 558)	P value
Age, years	61.8 ± 10.7	60.3 ± 11.8	64.2 ± 11.5	< 0.001
Sex, male, n (%)	49 (39.8)	52 (34.2)	209 (37.5)	0.618
BMI, kg/m^2^	21.8 ± 2.9	21.5 ± 2.6	21.0 ± 2.9	0.011
Ever smoker, n (%)	21 (17.1)	38 (25.0)	110 (19.7)	0.225
History of pulmonary TB, n (%)	35 (28.5)	36 (23.7)	187 (33.5)	0.053
Presence of bronchiectasis, n (%)	72 (58.5)	84 (55.3)	379 (67.9)	0.006
**Comorbidities, n (%)**				
COPD	8 (6.5)	3 (2.0)	47 (8.4)	0.021
Asthma	12 (9.8)	8 (5.3)	52 (9.3)	0.241
Malignancy	17 (13.8)	18 (11.8)	87 (15.6)	0.507
Diabetes mellitus	12 (9.8)	17 (11.2)	63 (11.3)	0.931
Chronic renal disease	2 (1.6)	4 (2.6)	11 (1.9)	0.814
Hypertension	24 (19.5)	30 (19.7)	125 (22.4)	0.691
GERD	6 (4.9)	6 (3.9)	28 (5.0)	0.885
Cardiovascular	11 (8.9)	6 (3.9)	41 (7.4)	0.215
**Symptoms, n (%)**				
Cough	53 (43.1)	42 (27.6)	219 (39.2)	0.012
Sputum	55 (44.7)	26 (17.1)	187 (33.5)	< 0.001
Hemoptysis	14 (11.4)	33 (21.7)	125 (22.4)	0.017
Dyspnea	7 (5.7)	10 (6.6)	59 (10.6)	0.122
Fever	9 (7.3)	10 (6.6)	12 (2.2)	0.002
Weight loss	0 (0.0)	2 (1.3)	15 (2.7)	0.140
**Radiographic type**				< 0.001
Fibrocavitary, n (%)	3 (2.4)	6 (3.9)	65 (11.6)	
Nodular bronchiectatic, n (%)	93 (75.6)	106 (69.7)	445 (79.7)	
Unclassifiable, n (%)	27 (21.9)	40 (26.3)	48 (8.6)	
No. of involved lobes	2.8 ± 1.7	2.6 ± 1.6	3.6 ± 1.6	< 0.001
Presence of cavity, n (%)	4 (3.6)	12 (7.9)	124 (22.2)	< 0.001
Severity of bronchiectasis^a^	2.6 ± 2.6	2.0 ± 1.8	3.0 ± 2.3	< 0.001
Positive AFB smear, n (%)	3 (2.4)	16 (10.5)	168 (30.1)	< 0.001
**Isolated organism, n (%)**				
*Mycobacterium avium*	78 (63.4)	69 (45.4)	252 (45.2)	
*Mycobacterium intracellulare*	26 (21.1)	37 (24.3)	162 (29.0)	
*Mycobacterium abscessus* complex^b^	11 (8.9)	37 (24.3)	82 (14.7)	
*Mycobacterium kansasii*	3 (2.4)	4 (2.6)	11 (2.0)	
Mixed infection^c^	5 (4.1)	5 (3.3)	51 (9.1)	

Categorical variables are expressed as number (%); continuous variables are expressed as mean ± standard deviation.

*AFB* acid-fast bacillus, *BMI* body mass index, *COPD* chronic obstructive pulmonary disease, *GERD* gastroesophageal reflux disease, *NTM* nontuberculous mycobacteria, *NTM-PD* nontuberculous mycobacterial pulmonary disease, *TB* tuberculosis.

^a^Assessed using a modified Reiff score.

^b^Includes *Mycobacterium abscessus* subspecies *abscessus and Mycobacterium abscessus* subspecies *massiliense.*

^c^More than two species among *Mycobacterium avium* complex, *Mycobacterium abscessus* complex, and *Mycobacterium kansasii.*

### Radiographic aggravation

**Figure 2:**
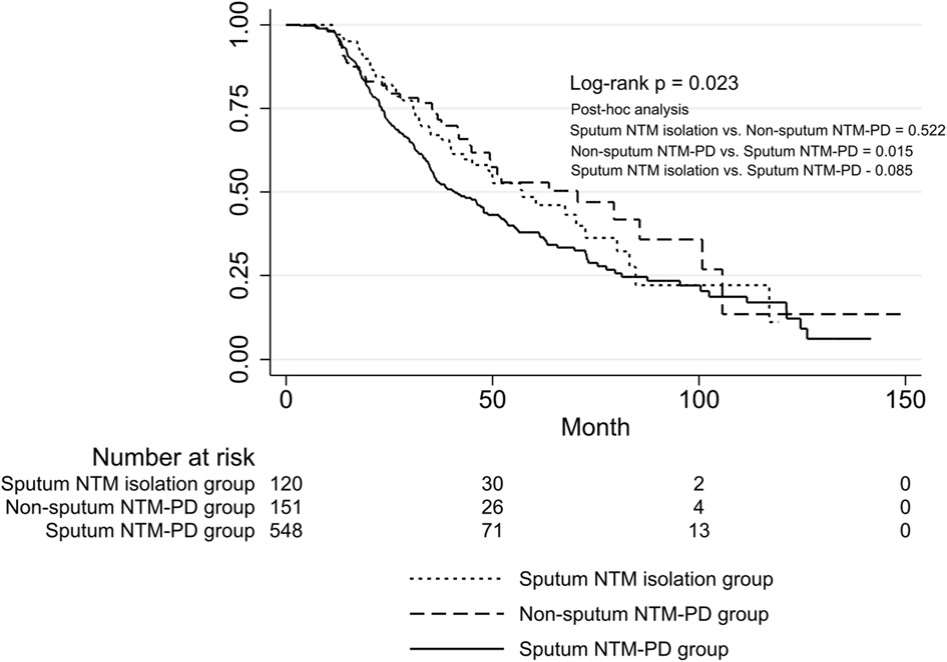
Comparison of time to radiographic aggravation for nontuberculous mycobacterial pulmonary disease (NTM-PD). Radiographic aggravation-free survival was significantly different between the groups (Log rank *P* = 0.023). Post-hoc analysis on the log-rank test with Bonferroni’s correction showed the significant difference of radiographic aggravation-free survival between the non-sputum and sputum NTM-PD groups. Patients with a single NTM isolation from sputum were designated as the sputum NTM isolation group, patients with positive results from bronchoscopic [e.g., bronchial washing or bronchoalveolar lavage (BAL)] or lung tissue specimens were designated as the NTM-PD group diagnosed using other methods (non-sputum NTM-PD group), and patients with NTM isolation at least two times from separately expectorated sputum samples were designated as the NTM-PD group diagnosed using sputum culture (sputum NTM-PD group).

During the follow-up period, 48 (39.0%) patients in the sputum NTM isolation group, 39 (25.7%) patients in the non-sputum NTM-PD group, and 179 (32.1%) in the sputum NTM-PD group showed radiographic aggravation. The time to radiographic aggravation was significantly different among the groups (log-rank test; p = 0.023). Post-hoc analysis on the log-rank test showed a significant difference between the non-sputum NTM-PD and the sputum NTM-PD group (p = 0.015) (Fig. 2). Cox proportional hazard regression was performed to determine factors associated with radiographic aggravation of NTM-PD. In the multivariable Cox proportional hazard regression, the sputum NTM-PD group remained independently associated with a higher risk of radiologic aggravation (aHR, 1.52; 95% CI, 1.06–2.19) relative to the non-sputum NTM-PD group after adjustment for age, sex, BMI, and radiographic type (Table 2).

**Table 2:** Results of Cox proportional hazards analysis for radiographic aggravation.

	Univariable		Multivariable	
	HR (95% CI)	*P*-value	Adjusted HR (95% CI)	*P*-value
**Group**				
Non-sputum NTM-PD group	Reference		Reference	
Sputum NTM isolation group	1.16 (0.76–1.77)	0.484	1.09 (0.70–1.69)	0.693
Sputum NTM-PD group	1.53 (1.08–2.17)	0.016	1.52 (1.06–2.19)	0.022
Age, year	1.00 (0.99–1.01)	0.915	1.00 (0.98–1.01)	0.323
BMI, kg/m^2^	0.89 (0.85–0.94)	< 0.001	0.90 (0.86–0.95)	< 0.001
Female	0.93 (0.76–1.27)	0.892	0.87 (0.65–1.15)	0.336
Ever smoker	0.99 (0.70–1.38)	0.935		
History of pulmonary TB	0.84 (0.64–1.10)	0.202		
**Radiographic type**				
Fibrocavitary	Reference		Reference	
Nodular bronchiectatic	0.91 (0.43–1.94)	0.811	1.06 (0.46–2.43)	0.888
Unclassifiable	0.45 (0.19–1.05)	0.066	0.63 (0.25–1.55)	0.314
No. of involved lobes^a^	1.19 (1.11–1.28)	< 0.001		
Presence of cavity^a^	1.66 (1.04–2.66)	0.033		
Positive AFB smear^a^	1.78 (1.31–2.42)	< 0.001		

*BMI* body mass index, *COPD* chronic obstructive pulmonary disease, *NTM* nontuberculous mycobacteria, *NTM-PD* nontuberculous mycobacterial pulmonary disease, *TB* tuberculosis.

^a^Not included in multivariable analysis owing to multicollinearity with Group factor.

### Initiation of treatment

**Figure 3:**
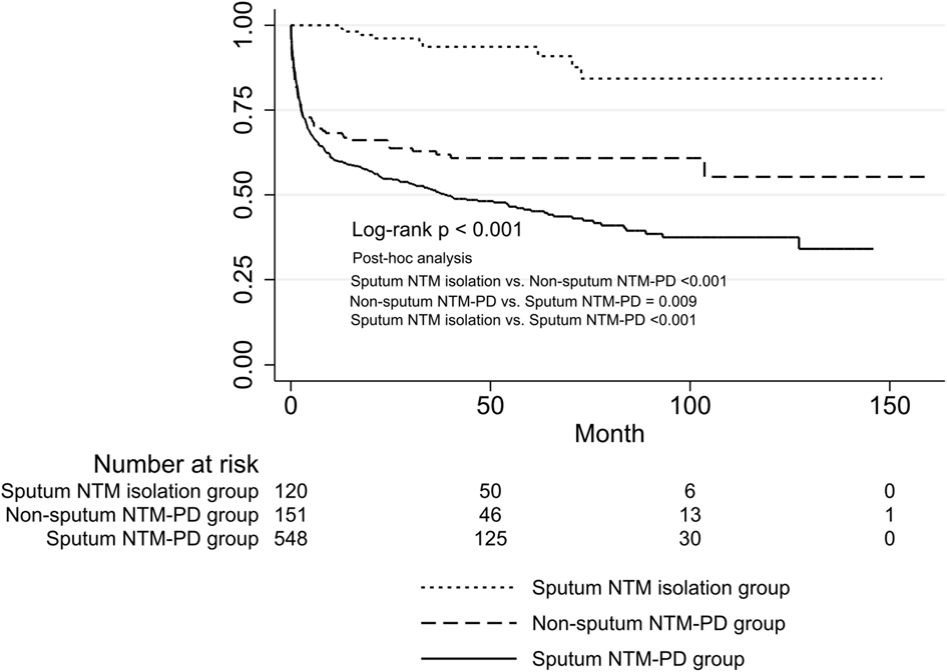
Comparison of time to initiation of treatment for nontuberculous mycobacterial pulmonary disease (NTM-PD). Treatment-free survival was significantly different between the groups (Log rank *P* < 0.001). Post-hoc analysis on the log-rank test with Bonferroni’s correction showed that treatment-free survival significantly increased in the order of the sputum NTM isolation, the non-sputum NTM-PD, and the sputum NTM isolation group. Patients with a single NTM isolation from sputum were designated as the sputum NTM isolation group, patients with positive results from bronchoscopic [e.g., bronchial washing or bronchoalveolar lavage (BAL)] or lung tissue specimens were designated as the NTM-PD group diagnosed using other methods (non-sputum NTM-PD group), and patients with NTM isolation at least two times from separately expectorated sputum samples were designated as the NTM-PD group diagnosed using sputum culture (sputum NTM-PD group).

Among all patients, nine (7.3%) in the sputum NTM isolation group, 300 (53.8%) in the sputum NTM-PD group, and 59 (38.8%) in the non-sputum NTM-PD group began therapy for NTM-PD after time zero. The time to initiation of therapy for NTM-PD was significantly different among the groups (log-rank test; p < 0.001). Post-hoc analysis on the log-rank test showed that the time to initiation of therapy significantly increased in the order of sputum NTM isolation, non-sputum NTM-PD, and sputum NTM isolation group (Fig. 3). Cox proportional hazard regression was performed to determine factors associated with the initiation of treatment for NTM-PD. In the multivariable Cox proportional hazard regression, the sputum NTM-PD group remained independently associated with a higher risk of initiation of treatment (aHR, 1.36; 95% CI, 1.01–1.84) relative to the non-sputum NTM-PD group after adjustment for age, sex, smoking status, BMI, presence of asthma, and radiologic type (Table 3).

**Table 3:** Results of Cox proportional hazards analysis for initiation of treatment.

	Univariable		Multivariable	
	HR (95% CI)	*P*-value	Adjusted HR (95% CI)	*P*-value
**Group**				
Non-sputum NTM-PD group	Reference		Reference	
Sputum NTM isolation group	0.16 (0.08–0.32)	< 0.001	0.16 (0.08–0.34)	< 0.001
Sputum NTM-PD group	1.46 (1.10–1.94)	0.009	1.36 (1.01–1.84)	0.045
Age, year	1.00 (0.99–1.01)	0.541	0.99 (0.99–1.01)	0.903
BMI, kg/m^2^	0.89 (0.86–0.93)	< 0.001	0.92 (0.88–0.96)	< 0.001
Female	0.72 (0.58–0.89)	0.002	0.88 (0.65–1.19)	0.406
Ever smoker	1.43 (1.12–1.83)	0.004	1.10 (0.79–1.52)	0.567
History of pulmonary TB	1.10 (0.88–1.37)	0.403		
COPD	1.36 (0.93–1.97)	0.108		
Asthma	0.56 (0.36–0.87)	0.009	0.63 (0.40–0.97)	0.038
**Radiographic type**				
Fibrocavitary	Reference		Reference	
Nodular bronchiectatic	0.25 (0.18–0.33)	< 0.001	0.31 (0.22–0.43)	< 0.001
Unclassifiable	0.19 (0.12–0.29)	< 0.001	0.33 (0.21–0.52)	< 0.001
No. of involved lobes^a^	1.20 (1.13–1.29)	< 0.001		
Presence of cavity^a^	4.78 (3.81–5.99)	< 0.001		
Positive AFB smear^a^	3.27 (2.64–4.06)	< 0.001		

*BMI* body mass index, *COPD* chronic obstructive pulmonary disease, *GERD* gastroesophageal reflux disease, *NTM* nontuberculous mycobacteria, *NTM-PD* nontuberculous mycobacterial pulmonary disease, *TB* tuberculosis.

^a^Not included in multivariable analysis owing to multicollinearity with Group factor.

## Discussion

This study investigated whether NTM-PD diagnosed using bronchoscopy or lung tissue biopsy not meeting sputum culture criteria in accordance with the ATS/IDSA guidelines exhibits the typical course of NTM-PD. We compared the disease progression of patients diagnosed with NTM-PD using bronchoscopy or lung tissue biopsy with those of sputum NTM isolation group and sputum NTM-PD group. There were significant differences between the non-sputum NTM-PD group and sputum NTM-PD group in terms of radiographic aggravation and initiation of treatment. However, no significant differences were observed between the non-sputum NTM-PD and sputum NTM isolation groups regarding radiographic aggravation.

In this study, the proportion of patients who received treatment in the sputum NTM-PD group was in line with previous studies investigating the natural course of NTM-PD^14,17^. Furthermore, the proportion of patients in the sputum NTM isolation group who received treatment was similar to that of a study that investigated the natural course of subjects with NTM isolates^17^. Post-hoc analysis on the log-rank test regarding initiation of therapy showed that the non-sputum NTM-PD group had longer treatment-free survival than the sputum NTM-PD group, while it was shorter than the sputum NTM isolation group. Likewise, the multivariable Cox analysis showed that the non-sputum NTM-PD group had a lower risk of treatment than the sputum NTM-PD group, while higher than the sputum NTM isolation group. In addition, post-hoc analysis on the log-rank test regarding radiographic aggravation showed that the non-sputum NTM-PD group had longer progression-free survival than the sputum NTM-PD group. Likewise, multivariable Cox analysis showed that the non-sputum NTM-PD group had a lower risk of treatment than the sputum NTM-PD group. Interestingly, there was no difference between the non-sputum NTM-PD group and the sputum NTM isolation group in radiographic aggravation. Physicians consider factors besides radiographic aggravation for initiation of treatment, which can be a reason for the different results between the radiographic aggravation and initiation of treatment. From a different perspective, the decision to initiate treatment for suspected NTM-PD might be influenced by the guidelines-based diagnosis. Considering this, the high risk of treatment in the non-sputum NTM-PD group may be attributed to an early confirmed diagnosis, unlike in the sputum NTM isolation group. Of course, caution is required when comparing the result of non-sputum NTM-PD group with the sputum NTM isolation group which may be composed of heterogeneous population including mild NTM-PD patients and participants without NTM-PD. Most importantly, our findings suggest that NTM-PD, diagnosed using bronchoscopy following insufficient results from sputum specimens, may be a milder disease than NTM-PD diagnosed using separately expectorated sputum, therefore showing slower disease progression.

Previous studies reported that NTM was isolated in a significant proportion of patients with underlying lung disease such as cystic fibrosis bronchiectasis and non-cystic fibrosis bronchiectasis^18–21^. Patients with underlying lung disease frequently have symptoms and radiographic findings that are difficult to distinguish from those of NTM-PD. Therefore, such patients are more likely to be perceived as having a severe disease despite the possibility of mild NTM-PD. In fact, the majority of patients enrolled in this study had a history of pulmonary TB or an underlying lung disease, such as bronchiectasis, which is difficult to differentiate from NTM-PD. Interestingly, patients in the sputum NTM-PD were more likely to have more extensive lobar involvement and more cavitary lesions than the other groups. Moreover, patients with sputum NTM-PD were more likely to have a positive AFB smear. AFB smear positivity and radiologic features such as the extent of disease and cavitary lesions are well-known factors related to the progression of NTM-PD^15,22–26^. The correlation between the diagnostic method and the factors suggests that the diagnostic method shares the characteristics of the factors. Detection of Mycobacterium and other bacteria with bronchoscopy was shown to be superior to that of sputum culture^8,9,27–29^. In accordance with these studies, our findings also suggest that bronchoscopic culture is more sensitive for detection of NTM strains than sputum culture, regardless of the bacterial burden and disease severity. Consequently, some of the patients diagnosed with bronchoscopy could have been over-diagnosed for NTM-PD despite mild disease state and small bacterial burden, due to ambiguous symptoms and high sensitivity.

In this study, low BMI and radiologic type were identified in the multivariable analysis as factors other than the diagnostic method associated with disease progression. Low BMI is associated with an increased risk of disease progression in NTM-PD^14,30–32^. Fibrocavitary radiographic type showed a higher risk for treatment than the other radiographic type. This is in line with previous studies that reported high mortality and high risk of treatment initiation of fibrocavitary-type disease^4,14,33^. In univariable Cox analyses, factors including AFB smear positivity, number of involved lobes, and presence of cavity were associated with an increased risk of disease progression. However, these factors were excluded from the multivariable Cox analyses because they could mitigate the impact of the diagnostic method due to their multicollinearity with the diagnostic method.

We investigated, for the first time, the prognosis of a large number of patients with NTM-PD diagnosed using bronchoscopy or lung tissue biopsy. To the best of our knowledge, no studies have evaluated the long-term prognosis of NTM-PD diagnosed using microbiologic methods other than sputum culture. In addition to Kaplan–Meier analysis with post-hoc analysis, Cox proportional hazards analysis was also used for the time of disease progression and adjustment for other factors.

The retrospective design of our study was one of its major limitations because a considerable number of patients could not be followed up for a longer period of time or evaluated for disease progression. Nevertheless, the median follow-up period was longer in our study than in other studies. Furthermore, the number of sputum cultures differed for each patient during the follow-up period due to the study design. Another limitation was the lack of standardization in the radiologic evaluation methods and protocols used for microbiologic confirmation. For example, patients were evaluated using CT scans from diverse vendors, and intervals of the radiological evaluations differed. In addition, radiological findings in NTM-PD fluctuate frequently, and interpreting the results of radiological aggravation should be performed with caution and considered together with results of treatment initiation. To avoid the effect of treatment on radiographic evaluation, we immediately censored the patients who initiated treatment from the analyses of radiographic aggravation. A large number of censored cases in radiographic aggravation analysis can be a limitation, so sensitivity analysis after excluding the cases was performed (Supplementary Table). Although there was no difference in the results regarding the diagnosis group, the composition of the radiological type differed from the primary analysis because the fibrocavitary type had substantial censoring due to initiation of treatment. The bias owing to the influence of this difference on other variables cannot be ignored. Lastly, identifying the natural course of the disease through our study may be distorted by lead time bias.

In conclusion, patients diagnosed with NTM-PD using bronchoscopic or lung tissue specimens, who do not meet sputum culture criteria, showed a lower risk of disease progression than patients diagnosed with NTM-PD based on sputum culture. Our findings suggest that the NTM-PD diagnosed using methods other than sputum culture may be a mild disease, not equivalent to typical NTM-PD. Further multicenter prospective studies are needed to investigate the long-term prognosis of NTM-PD according to the method of microbiologic diagnosis.

## Supplementary Information

Supplementary Table.
