# Supplementary material for: Prognosis of nontuberculous mycobacterial pulmonary disease according to the method of microbiologic diagnosis
Source: Sci Rep. 2021 Apr 13;11:8036. doi: 10.1038/s41598-021-87197-9 (PMC8044107; doi:10.1038/s41598-021-87197-9)
Supplement: Supplementary file 1 — Supplementary Table. [file 41598_2021_87197_MOESM1_ESM.docx]

**Supplementary table**

Baseline characteristics and clinical features of the patients after excluding the censored cases

| **Variables** | **Sputum NTM**  **isolation group**  **(n = 106)** | **Non-sputum**  **NTM-PD group**  **(n = 101)** | **Sputum**  **NTM-PD group**  **(n = 313)** | **P-value** |
| --- | --- | --- | --- | --- |
| Age, years | 62.3 ± 10.3 | 59.1 ± 11.3 | 64.6 ± 11.2 | <0.001 |
| Sex, male, n (%) | 40 (37.7) | 31 (30.7) | 101 (32.3) | 0.494 |
| BMI, kg/m2 | 21.8 ± 2.8 | 21.4 ± 2.5 | 21.5 ± 2.7 | 0.639 |
| Ever smoker, n (%) | 17 (16.0) | 20 (19.8) | 55 (17.6) | 0.774 |
| Presence of bronchiectasis | 69 (65.1) | 70 (69.3) | 230 (73.5) | 0.232 |
| History of pulmonary TB | 50 (29.9) | 31 (25.2) | 122 (32.5) | 0.313 |
| Comorbidities, n (%) |  |  |  |  |
| COPD | 7 (6.6) | 2 (1.9) | 21 (6.7) | 0.179 |
| Asthma | 12 (11.3) | 5 (4.9) | 39 (12.5) | 0.090 |
| Malignancy | 13 (12.3) | 10 (9.9) | 50 (15.9) | 0.289 |
| Diabetes mellitus | 9 (8.5) | 14 (13.9 | 34 (10.9) | 0.484 |
| Chronic renal disease | 2 (1.9) | 3 (2.9) | 7 (2.2) | 0.844 |
| Hypertension | 20 (18.9) | 18 (17.8) | 75 (23.9) | 0.331 |
| GERD | 4 (3.8) | 6 (5.9) | 17 (5.4) | 0.758 |
| Cardiovascular | 10 (9.4) | 1 (1.0) | 30 (9.6) | 0.006 |
| Radiological type |  |  |  | 0.011 |
| Fibrocavitary, n (%) | 2 (1.9) | 4 (4.0) | 10 (3.2) |  |
| Nodular bronchiectatic, n (%) | 85 (80.2) | 73 (72.3) | 270 (86.3) |  |
| Unclassifiable, n (%) | 19 (17.9) | 24 (23.8) | 33 (10.5) |  |
| No. of involved lobes | 2.9 ± 1.7 | 2.6 ± 1.5 | 3.4 ± 1.5 | <0.001 |
| Presence of cavity, n (%) | 3 (2.8) | 5 (4.9) | 20 (6.4) | 0.392 |
| Severity of bronchiectasis | 2.8 ± 2.7 | 2.2 ± 1.9 | 2.9 ± 2.3 | 0.047 |
| Positive AFB smear, n (%) | 2 (1.9) | 7 (6.9) | 59 (18.8) | <0.001 |

Results of Cox proportional hazards analysis for radiographic aggravation after excluding the censored cases

| **Multivariable Cox analysis** | **aHR for radiographic aggravation** | **95% CI** | ***P*-value** |
| --- | --- | --- | --- |
| Group |  |  |  |
| Non-sputum NTM-PD group | reference |  |  |
| Sputum NTM isolation group | 1.09 | 0.70-1.68 | 0.708 |
| Sputum NTM-PD group | 1.55 | 1.08-2.22 | 0.018 |
| Age, year | 0.99 | 0.98-1.01 | 0.383 |
| BMI, kg/m^2^ | 0.90 | 0.86-0.95 | <0.001 |
| Female | 0.90 | 0.64-1.27 | 0.562 |
| Ever smoker | 1.07 | 0.70-1.62 | 0.750 |
| Asthma | 0.84 | 0.56-1.25 | 0.381 |
| Radiological type |  |  |  |
| Fibrocavitary | reference |  |  |
| Nodular bronchiectatic | 1.09 | 0.48-2.51 | 0.831 |
| Unclassifiable | 0.63 | 0.25-1.58 | 0.328 |
